# Clinical trials in the pandemic age: What is fit for purpose?

**DOI:** 10.12688/gatesopenres.13146.1

**Published:** 2020-06-09

**Authors:** Dan Hartman, Penny Heaton, Nick Cammack, Ian Hudson, Shawn Dolley, Elena Netsi, Thea Norman, Trevor Mundel

**Affiliations:** 1 Bill & Melinda Gates Foundation, Seattle, Washington, 98109, USA; 2 Bill & Melinda Gates Medical Research Institute, Cambridge, MA, 02138, USA; 3 Wellcome Trust, London, NW1 2BE, UK; 4 Open Global Health, Inc, Arlington, Virginia, 22201, USA

**Keywords:** COVID-19, clinical trials, pandemic, SARS-CoV-2, real-world evidence, master protocol, pre-exposure prophylaxis, post-exposure prophylaxis, safety and efficacy

## Abstract

It is critical to ensure that COVID-19 studies provide clear and timely answers to the scientific questions that will guide us to scalable solutions for all global regions. Significant challenges in operationalizing trials include public policies for managing the pandemic, public health and clinical capacity, travel and migration, and availability of tests and infrastructure. These factors lead to spikes and troughs in patient count by location, disrupting the ability to predict when or if a trial will reach recruitment goals. The focus must also be on understanding how to provide equitable access to these interventions ensuring that interventions reach those who need them the most, be it patients in low resource settings or vulnerable groups.  We introduce a website to be used by The Bill & Melinda Gates Foundation, Wellcome Trust, and other funders of the COVID Therapeutics Accelerator that accept proposals for future clinical research. The portal enables evaluations of clinical study applications that focus on study qualities most likely to lead to informative outcomes and completed studies.

## Special communication

The coronavirus SARS-CoV-2, which causes the severe acute respiratory disease COVID-19, has currently spread to almost every country and is causing increasing morbidity and mortality around the world with as yet an unknown conclusion. It is causing huge strains on our health systems and devastating world economies. As countries and states/provinces in middle- and high-income countries wrangle for effective personal protective equipment and ventilators, lower-income countries are contemplating what will happen when the “COVID-19 wave” becomes their reality which they will face without many of these critical tools. This further drives the urgency to identify and repurpose pharmaceuticals for use in these countries for both prophylaxis and treatment. An unprecedented number of clinical trials have been registered for COVID-19 ^1^, yet no therapy has been recognized by the scientific community to be efficacious and have an appropriate benefit-risk profile.

COVID-19 studies will need to evaluate the efficacy of interventions against different aspects of the disease (for example pre- or post-exposure prophylaxis, mild/moderate, or preventing severe disease; therapeutics, vaccines or diagnostics). It is critical to ensure these studies provide clear and timely answers to the scientific questions most needed to guide us not only through the initial wave of this pandemic but towards scalable solutions that help re-establish day-to-day healthy living and economic well-being ^2^. There are significant challenges in operationalizing trials during a pandemic and with SARS-CoV-2, a novel virus, we are learning its natural history in real time. Factors including public policies for managing the pandemic, public health and clinical capacity, travel and migration, and availability of tests and infrastructure all lead to spikes and troughs in patient count by location. These disrupt the ability to predict when a trial will reach recruitment goals or to finish. For example, trial suspensions have begun to appear due to a lack of available patients in some locales ^3^. It may be that only a centralized multi-trial monitoring platform, powered by widely adopted and validated COVID-19 epidemic prediction algorithms ^4^, could effectively predict recruitment success across the globe as a pandemic progresses.

The rapid identification of effective COVID-19 therapies and vaccines requires informative trial outcomes derived from robustly designed studies using standardized approaches with high-quality data. Particularly in a pandemic setting, stakeholders should be able to have rapid access to de-identified patient-level data, through managed or open access, and to results of related trials, to inform the design of new and ongoing studies, whether interventional or observational in nature. Rapid access to high-quality data and results is also critical to help build and support new policy approaches as well as in maintaining and strengthening trust between participants, the public and the research community ^5^. Using standard platform-based approaches enables stakeholders to harmonize, clean, analyze and use data insights much faster, whilst maintaining scientific rigour. Adopting this type of pandemic-centric fit-for-purpose set of best practices, study quality assessments, and data approaches makes sense when there is no time for errors.

The many trials being established will place demands for access to COVID-19 patients and in some cases may exceed the capacity for clinical research, particularly recognizing the challenges of running clinical research in the middle of a pandemic, with cases peaking at different times around the globe. A new paradigm for conducting clinical trials is needed in which pandemic predictive models are used to direct clinical trial site preparation in future hot zones, further enabled through a pre-approved master protocol or smaller studies with aligned endpoints. This approach would sync clinical trials with the pandemic. As waves of the COVID-19 pandemic surge around the globe and impact health systems and populations, so too do waves of frenetic effort to save lives. In this context, physicians, study participants and their families may feel there is insufficient time and confusing information on which studies to join. Second order stakeholders—governments, funders, pharma, academia, contract research organizations—also must weigh, very quickly, which studies are most likely to provide necessary information. In one study, statisticians found that across 171 COVID-19 clinical trials, there were over 300 distinct primary endpoints registered by principle investigators ^6^. This fragmentation makes it very challenging to aggregate findings systematically toward impacting policy decisions. Standardized, open, high-quality endpoints and master protocols can resolve much of the confusion.

Furthermore, such a standardized approach with tools such as clinical trial management dashboards and remote monitoring will facilitate rapid initiation of the protocol throughout the world which may prove necessary as the pandemic wanes in some regions, but infections increases in others. If we employ sound science with efficient study designs, we will help get the right products, to the right people at the right dose (in the case of therapeutics) and the right time. Standardized, open, high quality data outputs will ensure we have comparable results across trials and will enable faster secondary analysis from more researchers, leading to more knowledge of epidemiology, disease course, and outcomes. These include both pre-specified interim and final data outputs, as well as protocols and standards used to collect the data where possible ^7^. We ought to be mindful of interpreting individual trial data in isolation; sharing outputs with the research community and stakeholders will help maintain the level of rigour and scrutiny that is required and ensure that early indicators and interim findings are appropriately interpreted and communicated. Anything less than best practice design and common data platform approaches will lead to wasted effort and more lives lost. The focus must be on delivering robust and timely answers to scientific questions that demonstrate the safety and efficacy characteristics of the intervention.

The focus must also be on understanding how to provide equitable access to these interventions ensuring that interventions reach those who need them the most, be it patients in low resource settings or vulnerable groups. Delivering those answers can be completed more quickly when enabled by secondary analysis of observational data, electronic medical records, and other real-world data and directly feed in to local, country-level decision making pathways. Solid answers can quickly result in policy and practice changes that benefit the health of the public and subsequently world economies and the many other aspects of our lives that have been upended by this pandemic.

 The COVID-19 pandemic and simultaneous flood of observational studies and clinical trials comes at an interesting time. There is increasing interest in why so many studies end with stakeholders and participants expecting greater impact. This is not a new problem, nor one specifically related to COVID-19. The failure rate for a drug progressing from a phase 1 study to launch hovers around 90%, and evidence abounds that poor trial design and/or data quality are significant contributing factors ^8, 9^. Informative studies provide robust clinical insight, a solid on-ramp to the next phase of development or a change in policy or standard of care, or to the decision not to progress. In 2019, Dr. Deborah Zarin and colleagues coined the term “uninformative trials” ^10^. “An uninformative trial is “one that provides results that are not of meaningful use for a patient, clinician, researcher, or policy maker.” ^10^ In the supplement to their paper in JAMA, they identified twelve ‘red flags’: qualities of clinical trials associated with uninformative trials. A year earlier, Dr. Trudie Lang’s Global Health Network convened a survey asking Low and Middle Income country-based (LMIC) researchers what parts of trials needed *critical help* towards improving methodology ^11^. Respondents coalesced around 27 factors in need of help. Meanwhile, other experts have published their own studies and guidance to address the same question ^12– 15^. Studies that failed to influence policy change or a confident next step in a go/no-go decision were commonly associated with factors such as lack of use of common endpoints, lack of conservatism in effect estimates, not using biostatistical simulation to derive proper sample sizes, using unduly restrictive inclusion criteria, and avoiding use of innovative trial designs.

Clinical trials are not the only source of data. Real-world data, largescale epidemiological studies, health systems research and social sciences all play an integral part in building a comprehensive evidence base. Each approach to data generation has its advantages and disadvantages, however it is important that all relevant data is made available in open science data models. To help establish standardized clinical endpoints and case definitions that help regulators and policy makers evaluate data and make decisions, current efforts have accelerated to develop a standard approach to convert clinical trial data to the most commonly used observational, real world evidence data models. For example, the Observational Health Data Science & Informatics (OHDSI) open science consortium is refining a US National Institute of Health approach to migrate data from the standard Clinical Data Interchange Standards Consortium (CDISC) Study Data Tabulation Model (SDTM) format to the OHDSI Observational Medical Outcomes Partnership (OMOP) Common Data Model (CDM) ^16, 17^. Global experts and implementers abound, and sustainability is strong—these are the most widely used standards globally for formatting trial data and storing observational data ^18– 20^. Wide adoption of one of these standards enabled one study to compile a virtual real-world evidence dataset with over 300,000 users of hydroxychloroquine in combination with azithromycin ^21^. Likewise, wide adoption of, for example, the same inclusion/exclusion criteria and primary endpoints could enable clinical or implementation science researchers to compile a systematic review with compelling evidence.

 A robust understanding of risk factors and best practices for informative trial design creates a need for a diagnostic guide to help relevant stakeholders assess whether a proposed study is likely to provide definitive answers and lead to implementable results. This guide can quickly identify studies that lack basic components, ‘flag’ studies that could be predicted to end uninformatively and guide these stakeholders towards studies designed to address questions in more definitive ways. One guide that is free and publicly available for COVID-19 studies is located at https://bit.ly/dat-covid-19, and we encourage all researchers to use this when designing COVID-19 related clinical studies. The guide will be used to screen and help applicants design their clinical trials for the recently announced COVID-19 Therapeutic Accelerator, (see https://www.therapeuticsaccelerator.org/). Faced with an influx of COVID-19 clinical research approval requests, researchers and funders could use such an assessment tool to help select the most appropriate studies, those more likely to definitively answer clinical questions.

These still early days of COVID-19 are an ideal time to implement trial assessment guides. In addition to guiding investigative teams to ensure well-designed studies that will give an informative answer, the responses to key questions will enable the broad range of study reviewers (i.e., funders, implementers, sponsors, journal editors, peer reviewers) to expedite and better inform their respective reviews. Best practice methods can include striving toward both informative results as well as data that is findable, accessible, interoperable and reusable. A level of rigor will help route sick patients into studies most likely to be informative and ultimately to therapies more likely to be part of an at-scale response.

The COVID-19 pandemic calls for fit-for-purpose solutions that will surely challenge business as usual practices. Considerations around study design, real-world evidence to optimize the study, research methods, centralized recruitment and quality monitoring, use of common endpoints and established protocols and availability of interim and final data output for secondary analysis all depend on a variety of variables, including the purpose of the study and the data involved. As such, there is no one-size-fits-all rule of thumb. It is the responsibility of funders, sponsors, regulators, IRBs and physicians to use our best efforts to design, analyze and communicate studies that will frequently and efficiently generate definitive results. With multiple vaccine candidates currently being developed and tested, the same considerations and best practices apply to therapeutic as to vaccine trials. Tools for evaluating designs and protocols as well as guiding toward best practice interoperable data platforms are urgently needed now to help identify appropriate therapies for COVID-19. In the process, such tools could teach the next generation of researchers how to design studies and generate data that will affect policy and practice change by integrating best practices for implementation research, health systems strengthening, biostatistics, epidemiology, participant engagement, and model-informed drug development. Adoption of well-governed, global master protocols and participation in consortia that could develop lasting standards will help now and in the future ^1, 22^. Our commonality of purpose in this moment of crisis can be transformative, not only for therapies rapidly identified to save lives but for research and business innovations that accelerate health and equity for those around the world needing it the most.

## Data availability

No data are associated with this article.
