# Supplementary material for: Clinical trials in the pandemic age: What is fit for purpose?
Source: Gates Open Res. 2020 Jun 9;4:58. [Version 1] doi: 10.12688/gatesopenres.13146.1 (PMC7324688; doi:10.12688/gatesopenres.13146.1)
Supplement: Peer review history

# Peer review: Reviewer response for version 1

**DOI:** 10.21956/gatesopenres.14337.r28905

**Published:** 2020-06-29

**Authors:** Moji Christianah Adeyeye

**Affiliations:** 1 National Agency for Food and Drug Administration and Control (NAFDAC), Abuja, Nigeria

The authors presented design, analyze and communication tools for clinical trials studies in COVID-19 pandemic that could generate informative and definitive real-world reusable data that will be fit-for purpose and which could affect policy and practice change. The approach could be transformative for identification of new therapies with positive outcomes.

Does the article adequately reference differing views and opinions?

Yes

Are all factual statements correct, and are statements and arguments made adequately supported by citations?

Yes

Is the Open Letter written in accessible language?

Yes

Where applicable, are recommendations and next steps explained clearly for others to follow?

Yes

Is the rationale for the Open Letter provided in sufficient detail?

Yes

Reviewer Expertise:

Early phase Quality-by-design drug product development, Drug product evaluation, Bioequivalence clinical trials, fit-for regulatory purpose driven research

I confirm that I have read this submission and believe that I have an appropriate level of expertise to confirm that it is of an acceptable scientific standard.

# Peer review: Reviewer response for version 1

**DOI:** 10.21956/gatesopenres.14337.r28907

**Published:** 2020-06-16

**Authors:** Ndansi Elvis Nukam

**Affiliations:** 1 Unite for Health Foundation, New York City, NY, USA

This is a well-written, needed, and useful summary description of the current status and perspective for conducting clinical trials during a pandemic. The strength of this article is that it addresses an interesting and timely question on how to deal with the challenges of conducting clinical trials during a pandemic, and finds a novel solution based on a carefully selected set of potential recommended actions. The authors highlighted some of the challenges of operationalizing trials during a pandemic and with SARS-CoV-2 clearly stating influencers such as public policy, public health and clinical capacity, travel and migration, and availability of testing and infrastructure. The article points out one of the major consequences of the aforementioned challenges which are the disruption in the ability to predict when a trial will reach recruitment goals or to finish.

 A relevant reflection on some set of best practices such as the use of a pandemic predictive model to direct clinical trial site preparation in future hot zones has been provided. The article also highlights the need for the use of standardized open, high-quality endpoints and master protocols as a means of resolving existing confusion of numerous distinct endpoints registered by different researchers during a trial. This is an approach towards innovating in clinical trial methodologies during a pandemic. By stating the challenges of conducting clinical trials during the COVID-19 pandemic, racing the need for a new paradigm for conducting clinical trials, and making suggestions on some best practices that can be used by researchers, the rationale for this article has been justified.

 Though the recommendations raised in this article are not all backed by evidence-based references, they are all interesting opinions that should be tested. For example, by raising the need for a diagnostic guide to help relevant stakeholders assess whether a proposed study is likely to provide definitive answers and lead to implementable results, the article tries to make recommendations that provide a baseline for further reflections.

 This article also serves as a trigger for further deep thinking and necessary action that could improve the clinical trial process during a pandemic. There is a need for further reflections in subsequent related articles on other important aspects of clinical trials during a pandemic such as communication and community engagement as well as the challenging issues of process and ethics.

 I have no major concern about the article.

Does the article adequately reference differing views and opinions?

Yes

Are all factual statements correct, and are statements and arguments made adequately supported by citations?

Yes

Is the Open Letter written in accessible language?

Yes

Where applicable, are recommendations and next steps explained clearly for others to follow?

Yes

Is the rationale for the Open Letter provided in sufficient detail?

Yes

Reviewer Expertise:

Public Health, Infectious diseases, Health Research Ethics, Clinical Trial Design, and Interpretation.

I confirm that I have read this submission and believe that I have an appropriate level of expertise to confirm that it is of an acceptable scientific standard.
